# Biophysical Micromixer

**DOI:** 10.3390/s90705379

**Published:** 2009-07-08

**Authors:** Chin-Tsan Wang, Yuh-Chung Hu, Tzu-Yang Hu

**Affiliations:** Department of Mechanical and Electromechanical Engineering, Center of Green Technology, National I-Lan University, I Lan, 26047, Taiwan; E-Mails: ychu@niu.edu.tw (Y.-C.H.); hour7593@yahoo.com.tw (T.-Y.H.)

**Keywords:** passive micromixer, biophysical micromixer

## Abstract

In this study a biophysical passive micromixer with channel anamorphosis in a space of 370 μm, which is shorter than traditional passive micromixers, could be created by mimicing features of vascular flow networks and executed with Reynolds numbers ranging from 1 to 90. Split and recombination (SAR) was the main mixing method for enhancing the convection effect and promoting the mixing performance in the biophysical channel. The 2D numerical results reveal that good mixing efficiency of the mixer was possible, with ε_mixing_ = 0.876 at Reynolds number ration Re_r_ = 0.85. Generally speaking, increasing the Reynolds number will enhance the mixing. In addition, the sidewall effect will influence the mixing performance and an optimal mixing performance with ε_mixing_ = 0.803 will occur at an aspect ratio of AR = 2. These findings will be useful for enhancing mixing performance for passive micromixers.

## 1. Introduction

Microfluidic systems have been widely applied in biochemical, biological and chemical analysis for their potential and advantages, such as the need for small amounts of sample and reagent, less time consumption, lower cost and high throughput. In addition, micromixers play a core role in many biochemistry and biomedical applications, such as analysis and synthesis of RNA/DNA, PCR amplification, and so on [1,2]. Different mixing principles and micromixer designs to enhance fluid mixing within the microchannel have been reported. Micromixers can be mainly categorized as active micromixers or passive micromixers. An active micromixer requires some external power to facilitate mixing. These external energy sources cause a periodic variation of flow rates, microimpellers, ultrasonic, and so on [3–5]. Their structures are often complicated and require a complex fabrication process as a transmission mechanism between the external energy source and the mixing chamber is needed. Nevertheless, in active micromixers the mixing time and microchannel length required for uniform mixing are generally less than those for passive micromixers. However, the requirement of external power makes them difficult to integrate with other microfluidic devices. The relatively higher power consumption and cost also make active mixers less attractive for disposable applications. Contrarily, passive micromixers including the biophysical micromixer addressed in this study are simple to operate because the mixing process occurs along with the structure change of microchannel, making them attractive and suitable for integration with other devices. A passive micromixer, however, acts upon the pressure drop of the fluids. The construction of a passive micromixer is simpler and its operation is less complicated than an active micromixer. Various mixing principles have been applied to passive micromixers to achieve higher mixing efficiencies. Interdigital multilamination, split and recombination, diffusion length decrease, vortex generation, and chaotic mixing [6–23] are just a few of them. An interdigital micromixer with alternating feed channels that periodically creates liquid multilamination was addressed and geometric focusing used to reduce the lamellae width and to accelerate mixing [7–9]. A split and recombination mixer addressed by Lee *et al*. [10] increases the contact surface area exponentially. Lin *et al*. [12] used a circular mixing chamber to generate a vortex and two inlet channels which were divided into eight individual channels. Stroock *et al*. [13] demonstrated that a staggered herringbone structure generates chaotic mixing. Two key issues in micromixer development usually concern a simple system design with a high mixing efficiency and effective techniques for examining mixing efficiency [24,25]. It is known that the flow inside microchannels is predominantly laminar and the Reynolds numbers are usually lower than 10. Therefore, mixing of fluids in microchannels is not easily implemented via mechanical stirring methods because of size limitations and fabrication difficulties [26]. Improvement in the flexibility and performance of microfluidic systems by incorporating a number of processes, including fluid handling and fluid motion, that cause rapid mixing on micro scale can become a challenging problem [27] In general, most traditional micromixers have been constructed with straight fluid channels and designed with a combination of fillisters and/or fold paths to enhance the mixing effect [28]. However, the design of a straight channel requires a longer length to achieve the goal of uniform mixing. Therefore, it is always associated with the problems of mixer size and full-field inspection. In addition, fluid mixing at the microscopic scale is far more difficult than that in macroscopic fluid devices. In a typical microfluidic device, viscosity dominates the flow and the fluid streams prefer to adopt laminar flow patterns. Thus, fluid mixing that mainly depends on molecular diffusion is very slow. To achieve optimal mixing, an efficient passive micro-mixer usually involves complex 3-dimensional geometries which are utilized to enhance the fluid lamination, stretching and folding. As mentioned above, mixing in the passive micromixer occurs with the diffusion of molecules in the microsystem and the process is so slow. Therefore, the complex geometry or long microchannel should be used for efficient mixing, which would cause a large pressure drop and difficulties in design and fabrication process. In order to overcome it, a novel passive micromixer utilizing a biophysical concept and possessing high flow uniformity and lower pressure drop would be addressed in this study.

## 2. Numerical Method

**Figure 1.**
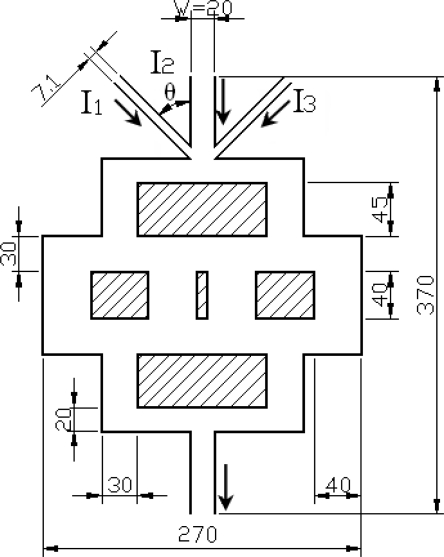
Prototype of biophysical micromixer (unit: μm), the arrows indicate the inlet and outlet flow direction.

In this study, a biophysical concept was applied to passive micromixers to promote mixing efficiency. In the biophysical micromixer shown in the Figure 1, the vertical width of channel would be gradually increased from 20 μm at the inlet to 40 μm at the middle section of the device, and then gradually decreased along the flow downstream to the outlet. At the inlet, the width of channels I_1_ and I_3_ is 7.1 μm, the width of channel I_2_ and the outlet channel is set as 20 μm. The total length of this device is 370 μm. The dimension of the biophysical micromixer is less than other traditional passive micro-mixers whose dimensions are even more than the order of mm and was made on purpose for this study because the effect of the mixing length is not the main objective in this experiment. Instead, this study focuses on the Reynolds ratio and mixing index. The Split andrecombination (SAR) micromixer used flat and slanted walls and embedded microchannels to split and recombine the flow for increasing the interaction. Oppositely, the biophysical micromixer would use variations of channels but without any plates along the flow downstream to the outlet. During the flow transmission process, the flow would be split first and then recombined with a flow motion similar to that of the SAR micromixer after the flow passes the middle section of the system to increasing interfaces exponentially by laminating the interfaces continuously along the channel. When solutions producing an interface between enters the channel, the interface is double that in one SAR mixing iteration. In addition, the convection effect would have another important role because a high flow uniformity and low pressure drop occur in a biophysical channel [29] on mixing and this is investigated in this study.

In this case, the fluid in the middle channel, labeled as Fluid I_2_, is assumed to contain a species such as protein or DNA, and the dimensionless concentration is set to unity for the Inlet 2 channel. In addition, the fluids in channels I_1_ and I_3_ are all the same and the concentrations would be assumed to be zero in name of pure water. The governing equations during the mixing process can be obtained by solving the continuity, momentum and diffusion equations, as follows:

(1) $$\nabla^{*}•{\vec{V}}^{*}=0$$

(2) $$\frac{{\partial \vec{V}}^{*}}{{\partial t}^{*}}+{\vec{V}}^{*}•\nabla^{*}{\vec{V}}^{*}=-\nabla^{*}p^{*}+\frac{1}{\text{Re}}\nabla^{*2}{\vec{V}}^{*}$$

(3) $$\frac{{\partial C}_{i}^{*}}{{\partial t}^{*}}+{\vec{V}}^{*}•\nabla^{*}C_{i}^{*}=\frac{1}{\text{Re}\mathrm{Sc}}\nabla^{*2}C_{i}^{*}$$

where, Re is the Reynolds number and defined as $\text{Re}=\frac{{\rho V}_{0}W}{\mu }$; and Sc, defined as $\mathrm{Sc}=\frac{\mu }{{\rho D}_{\mathrm{ij}}}$ is the Schmidt number to represent the ratio of viscosity effect to diffusion effect; W is the width of outlet channel, *V⃗* is the velocity vector, *t* is time, *p* denotes pressure, *C_i_* represents mole concentration, *V*_0_ is the characteristic velocity, μ is the fluid viscosity, ρ is the density of fluid, and *D_ij_* is the mass diffusivity. In addition, *Pe* is defined as $\mathrm{Pe}=\frac{\bar{U}D}{D_{\mathrm{AB}}}$ and is the Peclet number to show the ratio of convection effect to diffusion effect and will be investigated in this study.

The mixing performance was numerically simulated using a commercial CFD software package, CFD-ACE+. A multi-physics package based on the Finite-Volume method was applied. The program was run on a 2.4 Ghz Pentium IV processor with 1GB of RAM memory. Mesh-independent tests were performed before the studies. An upwind method for solving the multi-block unstructured grid of 15,000 cells was used as the 2D computational domain inside the micro-mixer. In addition, the grids ranging from 120,000 to 640,000 cells were used in 3D simulation for aspect ratio from 0.5 to 10.

The convergence criterion was assumed to be ± 10^−20^ for the residual of the discrete governing equation in the simulation. For the boundary conditions used in the simulation process, a constant inlet velocity calculated from a given Reynolds number ranging from 0.5 to 10 was used for steady-state analysis, and the outlet reference pressure was set as zero for guage pressure. Otherwise, the flow velocity, reference pressure, and concentration were all set to zero for initial conditions. Although the channel flow was laminar, a rather fine mesh was needed to account for the detailed features of the sorting mechanism. The time for each run spanned from 2 hours up to 3 hours.

The mixing flow was executed at Reynolds numbers, defined in terms of the inlet velocity and outlet channel width, ranging from 0.5 to 10. Convection produced in the biophysical micro-channels resulted in mixing.

Here a mixing efficiency ε_mixing_ defined in (4), was employed to find the optimal flow operation conditions:

(4) $$\varepsilon_{\text{mixing}}=1-\frac{1}{w}\int_{0}^{w}\left|\frac{X_{\text{Ax},\text{outlet}}-0.5}{X_{\text{Amax}}-0.5}\right|\mathrm{dx}$$

where X_Amax_ is the maximum mole fraction of Fluid A (fluid I_2_) and the value is unity. X_Ax,outlet_ is the mole fraction of Fluid A at outlet, and W = 20μm denotes the outlet width of the channel. As ε_mixing_ approaches 1, the mixing efficiency approaches a maximum. A larger value of ε_mixing_ corresponds to a better mixing performance.

## 3. Disscussions and Results

In this study, the operational Reynolds number defined in (5) was set in the range of Re = 0.5 to 10:

(5) $$\text{Re}=\frac{\rho U_{\mathrm{ave}}W}{\mu }$$

where ρ is the density of fluid. U_ave_ is the average velocity of the inlet channel; W, whose value is 20 μm, represents the width of inlet channel I_2_ and the outlet channel. μ is the dynamic viscosity of the working fluid.

To address the effect of the different inlet flow conditions on the mixing performance, a parameter denoted as Re_r_ defined in (6) was set for the Reynolds number ratio:

(6) $${\text{Re}}_{r}=\frac{{\text{Re}}_{1}+{\text{Re}}_{3}}{{\text{Re}}_{2}}$$

where, Re_1_ and Re_3_ are the Reynolds numbers for the side entrances of the inlet channel I_1_ and I_3_. Re_2_ is the inlet Reynolds number for the middle channel I_2_.

In addition, the aspect ratio, *AR*, ranging from 0.5 to 10 is defined in (7) and was investigated in order to study the side wall effects on mixing performance:

(7) $$\mathrm{AR}=\frac{D}{W}$$

where *D* is the depth of the channel and *W* is fixed at 20μm for the inlet mid-channel.

**Figure 2.**
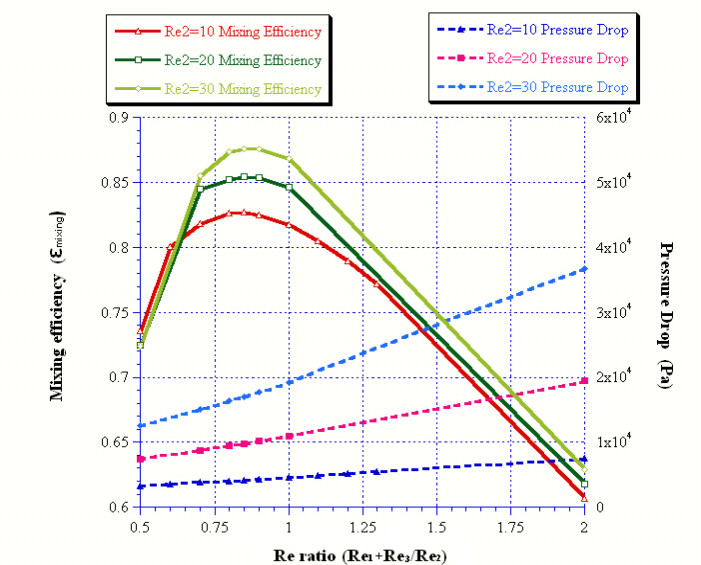
Reynolds number ratios versus the mixing efficiency and pressure drop.

In this study the flow mixing process were first observed at Reynolds numbers of the outlet channel ranging from 45 to 90. Some results with respect to Peclet number and Schmidt number for the studied cases are shown in Table 1 and addressed as follows:

First, the values of Peclet number, being larger than 5 for all studied cases, show that the convection effect was more dominant than the diffusion effect [2]. Second, the values of Schmidt number based on the studied cases was on the order of 3, and this indicates that the viscosity effect was more prominent than the diffusion effect [2]. Comparison between Peclet and Schmidt numbers for flow transmission process at all studied cases showed that convection was the main source of mixing and diffusion had less effect. In addition, the effect of the Reynolds number ratio on the mixing and pressure drop was studied and shown in Figure 2. The results are addressed as follows:

First, the optimal Reynolds number ratio was shown to be Rer = 0.85, because of its better mixing performance for different mid-channel inlet Reynolds numbers. Second, increasing the mid-channel inlet Reynolds number will enhance mixing. The mixing coefficient is ε_mixing_ = 0.876 at inlet Reynolds numbers of mid-channel Re_2_ = 30 and Rer = 0.85. This result indicates that this biophysical micromixer seems to be better than other passive micromixers in mixing over a mixing distance restricted to 370 μm. In addition, the pressure drop will also increase proportionally with the inlet Reynolds number.

**Figure 3.**
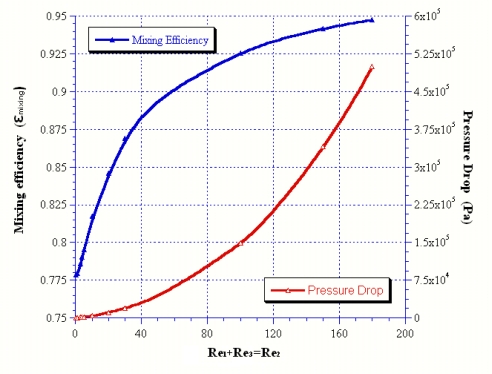
Reynolds number effect versus the mixing and pressure drop at Rer = 1.

Further investigation of the effect of Reynolds numbers on the mixing and pressure drop at Rer = 1(i.e., Re_1_+Re_3_ = Re_2_) is shown in Figure 3. The results show that increasing the inlet Reynolds number could definitely enhance mixing performance. The mixing coefficient will approach 0.95 when the Reynolds number of the inlet mid-channel is larger than 160. This finding shows that the Reynolds number positively affects mixing although it induces an increase in pressure drop.

**Figure 4.**
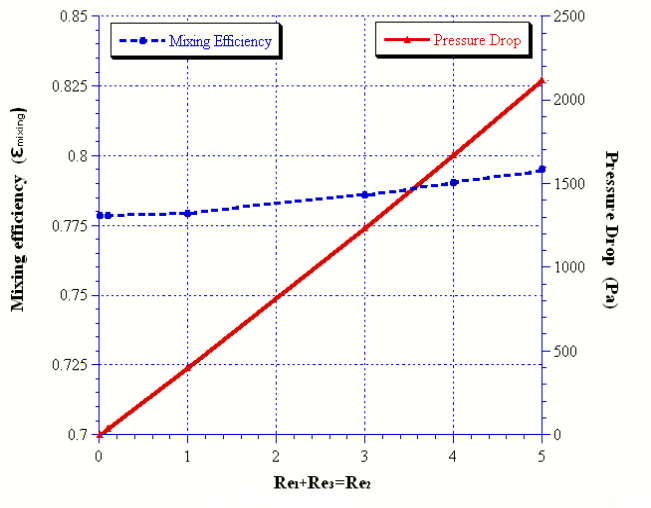
Reynolds number effect related to the mixing and pressure drop at Rer = 1.

Since the real flow velocity is relatively low in micro-channels, the effect of the Reynolds numbers ranging from 0.01 to 5 on the mixing and pressure drop was also investigated. The results of Figure 4 show that the mixing was about 0.778 at Reynolds number Re = 0.01, and increasing the Reynolds number will enhance mixing. In addition, the increase in pressure drop seems to be related to the Reynolds number.

Parameters such as the Reynolds number ratio and aspect ratio and their effect on mixing and pressure drop were further studied because finding the optimal Rer and AR is important for the operation of the micromixer. Hence, the Reynolds number ratio was decided and based on the variations of inlet Reynolds numbers from Re = 0.5 to 10 for the inlet channels. In addition, variations of aspect ratio were set as 0.5, 1, 2 and 10 for determining the side wall effect on mixing and pressure. The results, shown in the Table 2, are addressed as follows:

**Table 2.** Variations of mixing coefficient (ε_mixing_) and pressure drop (ΔP; unit: Pa) versus the Reynolds number ratio (Rer) and Aspect ratio (AR).

	Rer	0.5	0.85	1	2	1
	Re1	0.5	0.85	1	2	10
	Re2	1	1	1	1	10

ΔP	AR = 0.5	1890.52	2333.07	2522.87	3790.44	25999.58
ΔP	AR = 1	746.72	922.50	1891.34	1503.66	10908.40
ΔP	AR = 2	449.06	555.37	601.07	908.16	6915.66
ΔP	AR = 10	320.69	396.79	429.52	649.51	4956.02
ε_mixing_	AR = 0.5	0.72	0.79	0.78	0.61	0.76
ε_mixing_	AR = 1	0.73	0.79	0.78	0.59	0.80
ε_mixing_	AR = 2	0.69	0.80	0.80	0.67	0.85
ε_mixing_	AR = 10	0.73	0.79	0.79	0.62	0.83

First, the optimal Reynolds number ratio was Rer = 0.85, because of its outstanding mixing performance at different aspect ratios. Second, the side wall effect will influence the variations in pressure drop and mixing performance, and increasing the AR will also decrease the pressure. An optimal aspect ratio with highest mixing was found at AR = 2, which exhibited a good mixing for studied cases. In addition, the inlet angle of the side-channels and its effect on mixing and pressure was considered in the design of the micro-mixer. Hence, a variety of inlet angles of the side-channels, represented by θ, were executed with Reynolds number ratios ranging from Rer = 0.5 to 2 in the case of Re_2_ = 1 and its relationship with mixing performance and pressure drop are shown in Table 3.

The results of Table 3 show that a side-channel inlet angle of 30° was a better choice because it possesses a better mixing effect and has a lower pressure drop. These findings will be useful in the optimal design of a passive micromixer based on biophysical concepts in the further experimental study.

## 4. Conclusions

A novel micromixer design based on the biophysical concept was addressed in this study. The prototype was simple and possessed a better flow uniformity and lower pressure drop, so it could be expected to be useful to promote the mixing performance of passive micromixers when the mixing distance was restricted. Some numerical results related to mixing and pressure drop at variations of Reynolds number ratio, aspect ratio and side-channel inlet angle were executed and shown as following:

First, convection was the main source of mixing, whereas viscosity contributed only minutely, and diffusion the least. The split and recombination (SAR) was the main mixing method for enhancing the convection effect and promoting the mixing performance in biophysical micromixer. Second, the highest mixing coefficient with ε_mixing_ = 0.876 occurs at Reynolds number ratio, Rer = 0.85. Generally speaking, increasing the inlet Reynolds number will enhance mixing but also induce a pressure drop. Third, the side wall effect will influence the mixing performance and pressure drop. The aspect ratio of AR = 2 would be a better choice because it produces a good mixing coefficient of ε_mixing_ = 0.803 for the studied cases. When the aspect ratio was increased, the pressure drop would be decreased. Finally, the side-channel inlet angle of 30° is good choice because it produces better mixing and a lower pressure drop. These findings will be useful in the design of a optimal biophysical passive micromixer in further research.

## Figures and Tables

**Table 1.** Variations of Peclet number (Pe) and Schmidt number (Sc) versus Reynolds number.

**Re_outlet_**	45	54	57	60	90
**Pe**	40215.7	48258.84	50941.22	53620.92	80431.38
**Sc**	893.68	893.68	893.68	893.68	893.68

**Table 3.** Inlet angle of side-channel versus the mixing (ε_mixing_) and pressure drop (ΔP; unit: Pa) at variations of Reynolds number ratio ranging from Rer = 0.5 to 2 at the case of Re_2_ = 1.

	Rer = 0.5		Rer = 0.85		Rer = 1		Rer = 2	

Inlet angle of side channel, θ	ε_mixing_	ΔP	ε_mixing_	ΔP	ε_mixing_	ΔP	ε_mixing_	ΔP
90°	0.737	310.901	0.786	384.537	0.771	417.240	0.581	632.116
60°	0.738	300.758	0.791	371.628	0.776	403.076	0.580	609.910
45°	0.739	300.025	0.796	371.122	0.779	401.692	0.584	607.082
30°	0.738	300.781	0.803	371.240	0.790	402.526	0.589	607.998
0°	0.729	289.187	0.764	356.386	0.750	385.829	0.575	586.702
